# Current Evidence, Challenges, Opportunities, and Future Directions of Rotator Cuff Failure: A Systematic Review

**DOI:** 10.7759/cureus.111998

**Published:** 2026-07-03

**Authors:** Maithili V Potdar, Sandeep Shinde, Akshaya V Joshi, Sanjaykumar Patil

**Affiliations:** 1 Department of Musculoskeletal Sciences, Krishna College of Physiotherapy, Krishna Vishwa Vidyapeeth (Deemed to be University), Karad, IND; 2 Department of Obstetrics and Gynaecology, Krishna Institute of Medical Sciences, Krishna Vishwa Vidyapeeth (Deemed to be University), Karad, IND

**Keywords:** arthroscopic rotator cuff repair, conservative management, physical therapy, rotator cuff failure, rotator cuff tear

## Abstract

Rotator cuff tears (RTs) are among the most prevalent musculoskeletal conditions worldwide, imposing substantial burdens of chronic pain, functional limitation, and socioeconomic cost. The underlying pathophysiology is multifactorial, encompassing progressive mechanical attrition, tendon degeneration, and irreversible biological deterioration, including muscle atrophy, fatty infiltration, and altered glenohumeral kinematics that may ultimately culminate in cuff tear arthropathy. The central clinical challenge lies in stratifying patients appropriately and selecting the optimal treatment strategy before irreversible structural compromise renders either conservative or surgical intervention ineffective. This systematic review comprehensively synthesizes the available evidence on rotator cuff failure, evaluating its etiology, natural history, diagnostic accuracy of modern imaging modalities, and long-term functional outcomes, with direct comparison of conservative versus surgical management strategies and an assessment of emerging rehabilitation adjuncts. Adhering strictly to the Preferred Reporting Items for Systematic Reviews and Meta-Analyses (PRISMA) 2020 guidelines, a systematic search was performed across PubMed, Google Scholar, Scopus, and the Cochrane Library. Eligible studies included randomized controlled trials (RCTs) and cohort studies of adult patients with confirmed rotator cuff pathology diagnosed by MRI or high-resolution ultrasonography. Data extraction prioritized validated functional shoulder outcome scores. Methodological quality was assessed using the Cochrane Collaboration Risk of Bias 2 (RoB2) tool for RCTs and the Risk of Bias-I (ROBINS-I) tool for non-randomized observational studies. Twelve studies meeting full eligibility criteria were included. Multi-center RCTs and cohort studies demonstrate equivalent functional outcomes between conservative care and surgical repair for small-to-medium degenerative tears. Longitudinal cohort data identify a critical dose-effect threshold at 16 physical therapy sessions, beyond which further functional benefit plateaus. Arthroscopic repair delivers outcomes equivalent to open techniques with substantially reduced surgical morbidity. Emerging adjuncts, including pulley exercises at six weeks post-repair, blood flow restriction training, ultrasound-guided dry needling, and asynchronous telemedicine, demonstrate significant improvements in pain modulation, range of motion, and therapeutic adherence. Optimal management of rotator cuff failure demands an individualized, milestone-driven approach. Structured conventional therapy constitutes an evidence-supported first-line intervention for chronic degenerative tears; however, patients who fail to achieve functional milestones after conservative treatment require a timely transition to arthroscopic evaluation to prevent irreversible structural decline. Surgical approach, suture anchor technique, tissue biology, and postoperative protocol adherence are each determinative of repair success. Integration of advanced biological, digital, and remote rehabilitation modalities further bridges the gap between radiographic severity and real-world patient functionality.

## Introduction and background

The shoulder joint represents one of the most mechanically sophisticated structures in the human musculoskeletal system, and its extraordinary range of motion is critically dependent on the dynamic integrity of the rotator cuff. The rotator cuff is a complex musculotendinous unit comprising the supraspinatus, infraspinatus, teres minor, and subscapularis muscles [1]. Working in precise coordination, these muscles generate balanced compressive forces that dynamically center the humeral head within the glenoid fossa, effectively counterbalancing the powerful upward shear force exerted by the deltoid during arm elevation and enabling the full spectrum of shoulder kinematics [1,2].

When this sophisticated system fails due to degenerative attrition, acute traumatic disruption, or the insidious combination of both, the consequences extend far beyond focal tendon pathology. Patients may experience progressive functional disability, persistent nocturnal pain, diminished quality of life, and, if left untreated, potentially irreversible structural deterioration of the glenohumeral joint [1,2].

Rotator cuff tears (RTs) represent one of the most prevalent musculoskeletal pathologies encountered in clinical and surgical practice worldwide, with population-based imaging studies revealing a prevalence exceeding 20% in the general adult population, rising dramatically with advancing age [3]. Epidemiological data suggest that by the seventh decade of life, full-thickness tears are detectable in more than 40% of individuals undergoing shoulder MRI [4]. Crucially, a substantial proportion of these tears are initially asymptomatic, creating a deceptive and clinically perilous trajectory. Silent structural damage accumulates as biological degeneration progresses until a critical threshold is crossed and symptoms emerge, frequently accompanied by significant tendon retraction, advanced fatty infiltration, and muscle atrophy, all of which substantially complicate and may even preclude successful management [1,2].

Understanding why rotator cuff failure occurs requires an examination of both the intrinsic and extrinsic mechanisms that progressively undermine tendon structural integrity. Intrinsically, the supraspinatus tendon, by far the most commonly affected structure, is subject to a critical zone of relative avascularity approximately 1 cm proximal to its insertion on the greater tuberosity of the humerus [3]. This hypovascular “critical zone” exhibits markedly impaired cellular repair capacity due to reduced oxygen tension and diminished nutrient delivery, rendering it uniquely vulnerable to the cumulative microtrauma associated with repetitive shoulder loading throughout life [3]. Age-related changes in collagen cross-linking progressively reduce tendon viscoelasticity and tensile strength, while declining tenocyte populations impair the tissue's capacity for self-renewal and repair. At a systemic level, conditions including type 2 diabetes mellitus, hypercholesterolemia, and tobacco smoking have each been independently associated with accelerated tendon degeneration, implicating metabolic dysregulation as a critical upstream driver of structural deterioration [3,4].

Extrinsically, repetitive overhead activity, whether occupational (construction, painting, manual labor) or athletic (swimming, throwing sports, and racket sports), generates cyclical mechanical impingement of the supraspinatus tendon beneath the coracoacromial arch [3]. Over time, these repeated compressive and shear stresses initiate a predictable cascade of microstructural damage. Focal collagen fiber disruption progresses from partial-thickness articular-surface or bursal-surface tears to full-thickness defects and, in the absence of timely intervention, ultimately to massive, multi-tendon, irreparable tears that fundamentally alter glenohumeral biomechanics [5].

The biological consequences of an established full-thickness tear are progressive and, beyond a critical threshold, irreversible. Loss of homeostatic mechanical tension from tendon retraction initiates a degenerative cascade within the muscle belly. This includes progressive muscle atrophy and advanced fatty infiltration, classically quantified via the Goutallier system (Grades 0-4) [6]. Crucially, fatty infiltration exceeding Grade 2 and severe atrophy (indicated by a positive tangential sign) serve as primary predictors of poor structural outcomes following surgical repair, as the biological substrate required for successful tendon-to-bone healing becomes fundamentally compromised [7,8].

Biomechanically, a massive tear disrupts the glenohumeral force couple, allowing the unopposed deltoid to drive superior migration of the humeral head [6]. This pathological shift is marked by a narrowing of the acromiohumeral interval (AHI) below the normal 6 mm threshold. Chronic articulation against the coracoacromial arch triggers irreversible joint destruction, including acromial "acetabularization" and humeral "femoralization." These end-stage structural changes, categorized from Grade 1 to 5 via the Hamada grading system, represent advanced cuff tear arthropathy and signal severe joint failure [6].

Against this backdrop of escalating and potentially irreversible pathology, the central clinical imperative is timely and individualized intervention. The natural history of RTs is fundamentally one of progression. Longitudinal imaging studies consistently demonstrate that asymptomatic partial-thickness tears frequently evolve into full-thickness defects over time, smaller full-thickness tears tend to enlarge, and tear enlargement is directly associated with the development of fatty infiltration, muscle atrophy, and symptom onset [3,4,7]. These findings establish the existence of a critical and finite “window of opportunity” for intervention: a period during which structural damage has not yet exceeded the threshold of biological reparability and during which timely treatment, whether conservative or surgical, retains the capacity to restore functional anatomy and halt the degenerative cascade [3,4,7].

The diagnosis of rotator cuff pathology has evolved substantially beyond clinical examination alone. While provocative clinical tests, including the empty can test and drop arm test, retain diagnostic value as accessible first-line screening tools, definitive characterization of tear morphology, tear size, degree of tendon retraction, and tissue quality for preoperative planning now depends on advanced imaging modalities [2]. High-resolution musculoskeletal ultrasonography provides a dynamic, real-time, and cost-effective assessment, with sensitivity and specificity approaching those of MRI for the detection of full-thickness tears. Additionally, it permits functional evaluation during shoulder movement. MRI, particularly when combined with arthrographic enhancement, offers superior soft-tissue contrast resolution, enabling detailed characterization of partial-thickness tears, labral pathology, bone marrow edema, and muscle quality through standardized Goutallier grading and muscle-volume assessment criteria [4].

Management pathways are correspondingly nuanced. Conservative strategies, including structured physical therapy, activity modification, nonsteroidal anti-inflammatory medications, and judicious subacromial corticosteroid injections, remain the evidence-based first-line intervention for chronic degenerative tears in appropriately selected patients [3,4]. However, the literature increasingly recognizes that conservative management is not without risk when pursued without clearly defined milestones or temporal boundaries. Indefinitely prolonged nonoperative treatment in patients with progressive structural damage may delay surgical intervention beyond the point of reparability, allowing fatty infiltration and muscle atrophy to progress to irreversible stages that fundamentally compromise both the feasibility and long-term durability of subsequent surgical repair [4]. For acute traumatic tears, high-demand active patients, and individuals who fail structured conservative treatment with demonstrable structural progression, arthroscopic repair has emerged as the dominant surgical standard, delivering functional outcomes comparable to those of traditional open techniques while offering reduced perioperative morbidity, faster recovery, and enhanced postoperative rehabilitation opportunities [5].

**Figure 1:**
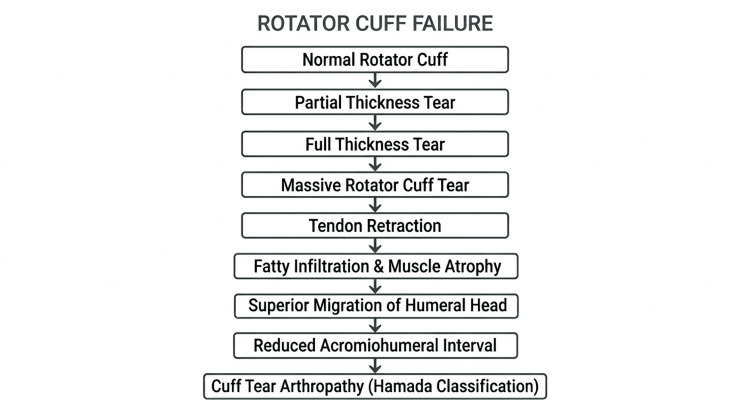
Clinical progression of rotator cuff failure from partial-thickness tear to cuff tear arthropathy (Hamada classification). The figure was created manually using the built-in feature, i.e., Microsoft Word SmartArt feature, which is a standard graphic design tool available within Microsoft Word for creating flowcharts and diagrams. No artificial intelligence (AI)-assisted image generation or editing features were used in the preparation of the figure.

This systematic review comprehensively addresses the multifaceted clinical challenge of rotator cuff failure by synthesizing the available evidence across its full spectrum. The specific objectives are to: (1) analyze the biomechanical and biological mechanisms underlying rotator cuff degeneration and repair failure, (2) evaluate the diagnostic accuracy of contemporary imaging and clinical assessment methods, (3) compare the long-term functional outcomes of conservative and surgical management strategies, and (4) identify current challenges, key prognostic determinants, and future research priorities to facilitate individualized patient care and optimize clinical outcomes. The step-by-step clinical and radiographic progression from an initial partial-thickness tear to end-stage cuff tear arthropathy is systematically outlined in Figure 1.

## Review

Methodology

Study Design

To ensure maximum clarity and scientific integrity, this systematic review adhered to the Preferred Reporting Items for Systematic Reviews and Meta-Analyses (PRISMA) guidelines for data collection and reporting [9].

Search Strategy

A comprehensive and systematic search was conducted across core electronic databases, including PubMed, Medical Literature Analysis and Retrieval System Online (MEDLINE), and Physiotherapy Evidence Database (PEDro). Appropriate keywords and Boolean operators (AND, OR) were used to refine the search. The keywords included “rotator cuff tear,” “massive rotator cuff tear,” “cuff tear arthropathy,” “Hamada classification,” “Goutallier grading,” “MRI shoulder,” “high-resolution musculoskeletal ultrasonography,” “arthroscopic rotator cuff repair,” “conservative management,” “physical therapy,” “blood flow restriction,” “dry needling,” and “telemedicine rehabilitation.” The literature search was restricted to English-language articles, with no date restrictions applied to capture the complete historical and contemporary evidence base from 2015 to 2026.

Inclusion Criteria

Studies were eligible for inclusion if they met the following pre-specified criteria: primary research designs consisting of randomized controlled trials (RCTs) or cohort studies; adult participants (>18 years) presenting with verified rotator cuff pathology, whether symptomatic or asymptomatic, spanning partial-thickness to massive irreparable tears; and publication in the English language between 2015 and 2026.

Exclusion Criteria

Studies were excluded if they: (1) focused primarily on non-cuff-related shoulder pathologies (isolated adhesive capsulitis, glenohumeral osteoarthritis without cuff involvement, or acute proximal humeral fractures), (2) enrolled patients with confounding systemic inflammatory or neurological conditions including rheumatoid arthritis, brachial plexus injuries, cervical radiculopathy, or isolated suprascapular neuropathy, (3) addressed revision surgery without specifically analyzing mechanisms of primary surgical failure, (4) represented low-evidence publication types including narrative reviews without systematic search methodology, editorials, conference abstracts, or expert opinion papers, or (5) reported pooled data from heterogeneous orthopedic injury populations without extractable rotator cuff-specific subgroup analyses.

Quality Assessment

Methodological quality and risk of bias were appraised using validated, study-specific tools tailored to the varying research designs. For the included RCTs, internal validity was examined using the Cochrane Collaboration’s risk of bias 2 (RoB 2) tool [10]. This instrument allowed for a systematic evaluation of critical methodological domains, namely the randomization process, deviations from intended interventions, missing outcome data, outcome measurement, and the selection of reported results. For the included cohort study, internal validity was assessed using the Risk Of Bias In Non-randomized Studies of Interventions (ROBINS-I) tool. This tool provides a structured evaluation of key methodological domains, including bias due to confounding, participant selection, classification of interventions, deviations from intended interventions, missing data, outcome measurement, and selective reporting of results [11].

Results

**Figure 2:**
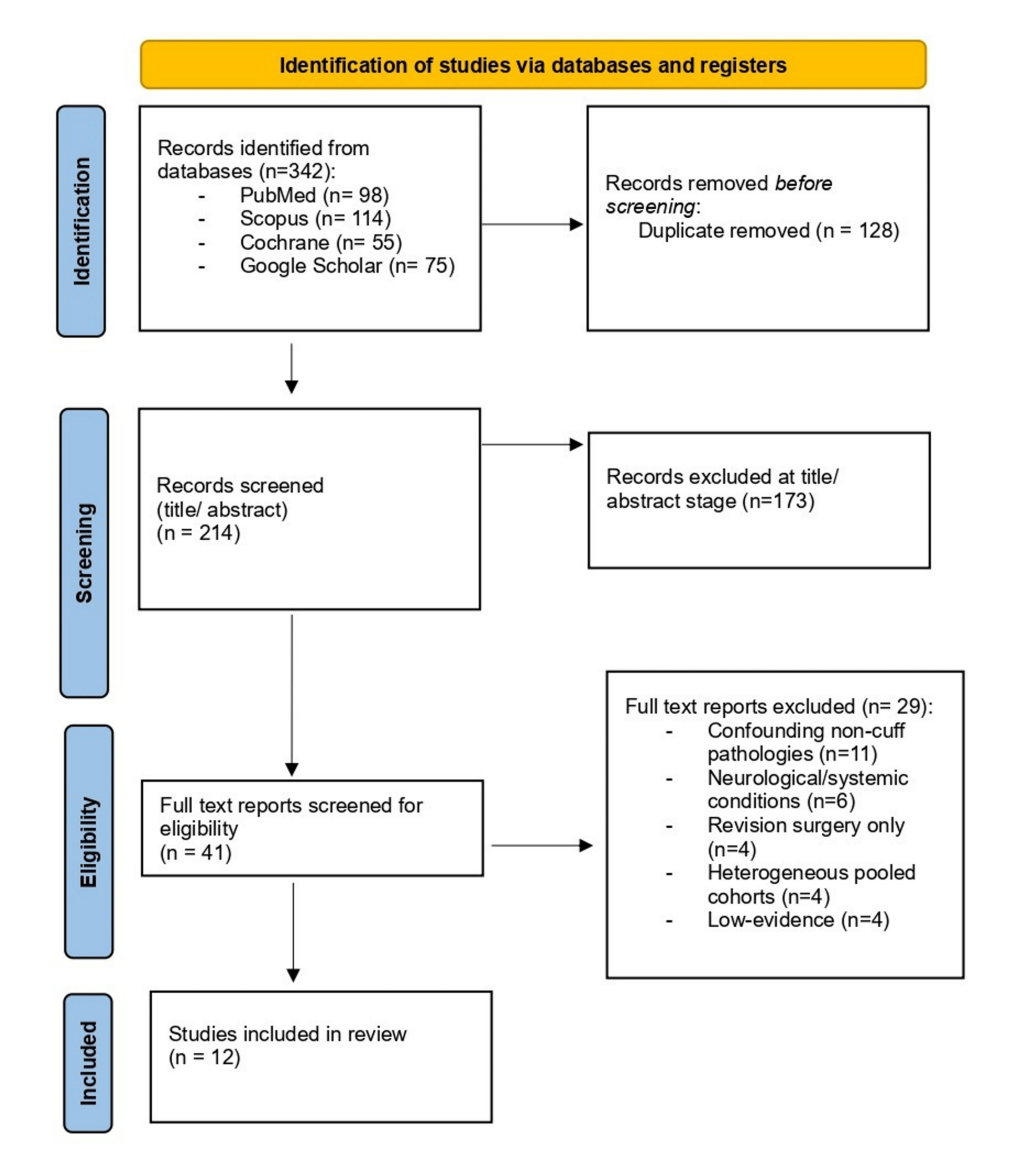
PRISMA flowchart. PRISMA: Preferred Reporting Items For Systematic Reviews and Meta-Analyses

Twelve primary studies met the full pre-specified eligibility criteria for inclusion in this systematic review. These comprised 12 RCTs or prospective randomized trials and one retrospective cohort study, collectively enrolling 1,101 participants across a diverse range of institutional and geographic settings. Study publication dates ranged from 2010 to 2026, reflecting both the established evidence base and the most contemporary emerging literature. Figure 2 shows the PRISMA flowchart.

Table 1 presents a comprehensive structured summary of study designs, sample sizes, primary outcome measures, key results, and conclusions.

**Table 1:** Summary of 12 included studies evaluating rotator cuff management: study design, sample size, primary results, outcome measures, and conclusions. CMS: Constant-Murley Score; VAS: Visual Analog Scale; MRI: magnetic resonance imaging; UCLA: University of California Los Angeles Shoulder Rating Scale; TENS: transcutaneous electrical nerve stimulation; WORC: Western Ontario Rotator Cuff Index; ASES: American Shoulder and Elbow Surgeons Score; SANE: single assessment numeric evaluation; SST: simple shoulder test; ROM: range of motion; OSS: Oxford Shoulder Score; QOL: quality of life; SPADI: Shoulder Pain and Disability Index; DASH: Disabilities of the Arm, Shoulder and Hand; PPT: pressure pain threshold; ASES: American Shoulder and Elbow Surgeons Score; NRS: Numerical Rating Scale; EQ-5D-5L: EuroQol Five-Dimension Five-Level Questionnaire; CPM: conditioned pain modulation; Quick DASH: Quick Disabilities of the Arm, Shoulder and Hand

Author (year)	Total participants	Outcomes measures	Intervention	Results	Conclusion
Kukkonen et al. (2015) [12]	180 (mean age: 65 years)	Primary outcome: Constant-Murley Score (CMS) Secondary outcomes: Visual Analog Scale (VAS) for pain, patient satisfaction, rotator cuff integrity/tear size on imaging, cost of treatment	Group 1: Physiotherapy only Group 2: Acromioplasty + physiotherapy Group 3: Rotator cuff repair + acromioplasty + physiotherapy	At the 2-year follow-up, all three treatment groups demonstrated significant improvement in shoulder function, with mean constant score improvements of 18.4 points in the physiotherapy group, 20.5 points in the acromioplasty plus physiotherapy group, and 22.6 points in the rotator cuff repair group. However, no statistically significant differences were found between the groups (p = 0.38). Similarly, there were no significant differences in VAS pain scores (p = 0.45) or patient satisfaction (p = 0.28). Although the rotator cuff repair group showed significantly less tendon tear progression, with a mean tear size of 4.2 mm compared to 11.0 mm in the non-repair groups (p < 0.01), surgical interventions were associated with substantially higher treatment costs than physiotherapy alone.	Conservative treatment is a reasonable first-line treatment for symptomatic nontraumatic supraspinatus tears in older adults.
Lambers Heerspink et al. (2015) [13]	56 patients (mean age-Surgery: 61.9 ± 7.0 year; conservative: 63.0 ± 8.2 year)	CMS, VAS Pain, VAS disability, magnetic resonance Imaging (MRI)	Surgical Group: Rotator cuff repair followed by postoperative rehabilitation Conservative Group: Physiotherapy-based rehabilitation program without surgery	At the 12-month follow-up, both treatment groups showed improvement in shoulder function, with the surgical group achieving a mean CMS of 81.9 ± 15.6 compared with 73.7 ± 18.4 in the conservative treatment group. However, this difference was not statistically significant (p = 0.08). Despite similar overall functional outcomes, patients who underwent surgical repair experienced significantly less pain (VAS, p = 0.04) and disability (VAS, p = 0.02) than those managed conservatively. Furthermore, the subgroup analysis revealed that patients with an intact repaired tendon had significantly superior functional outcomes, with a mean CMS of 88.5 ± 6.2, compared with 73.7 ± 18.4 in the conservative treatment group.	Surgery improved pain and disability, but not overall functional outcome, compared with conservative treatment at one year.
Analan et al. (2015) [14]	22 patients (mean Age: 52.9 ± 11.1 years, 57.1 ± 15.0 years)	VAS, CMS, University of California Los Angeles Shoulder (UCLA) Score, Isokinetic Strength	True Ultrasound Group: Continuous therapeutic ultrasound + hot pack + transcutaneous electrical nerve stimulation (TENS) + supervised exercise program Sham Ultrasound Group: Sham ultrasound + hot pack + TENS + supervised exercise program	Both the therapeutic ultrasound and sham ultrasound groups showed significant improvements in pain, shoulder function, and external rotator muscle strength following treatment. However, no significant differences were found between the groups, suggesting that therapeutic ultrasound provided no additional benefit beyond the standard physiotherapy program.	Therapeutic ultrasound offered no additional benefit beyond standard physiotherapy.
Baumgarten et al. (2016) [15]	53 patients (mean age: 27 pulley, 26 non-pulley)	Western Ontario Rotator Cuff Index (WORC), American Shoulder and Elbow Surgeons (ASES) Score, Single Assessment Numeric Evaluation (SANE), Shoulder Activity Level Simple Shoulder Test (SST), range of motion (ROM) Scapular substitution shoulder strength (scaption strength)	Pulley Group: Standard postoperative rehabilitation with shoulder pulley exercises initiated six weeks after rotator cuff repair. Non-Pulley Group: Standard postoperative rehabilitation without pulley exercises.	At the 52-week follow-up, both the pulley and non-pulley rehabilitation groups showed significant improvements in shoulder function, patient-reported outcomes, range of motion, strength, and scapular mechanics, with significant gains observed in WORC, ASES, SST, and SANE scores, as well as shoulder flexion, abduction, and external rotation (all p < 0.0001). However, no significant differences were found between the two groups in functional outcomes, range of motion, strength, shoulder activity level, or scapular substitution, indicating that the addition of pulley exercises did not provide any superior clinical benefit.	Pulley exercises initiated six weeks postoperatively were safe and produced outcomes comparable to rehabilitation without pulleys.
Carr et al. (2017) [16]	273 patients (mean age: ≥50 years) (mean age: approximately 64 years)	Oxford Shoulder Score (OSS), MRI integrity, quality of life (QOL), complications and adverse events, cost-effective analysis	Arthroscopic Group: Arthroscopic rotator cuff repair. Open Group: Open rotator cuff repair.	At the 24-month follow-up, both arthroscopic and open rotator cuff repair groups demonstrated substantial improvements in OSS, increasing from 26.3 to 41.7 and from 25.0 to 41.5, respectively, with no significant difference between the two techniques (p = 0.452). Re-tear rates were comparable between the arthroscopic (46.4%) and open repair (38.6%) groups, and complication rates and cost-effectiveness were similar. Patients with successfully healed tendon repairs experienced the greatest functional improvement, whereas those with re-tears or irreparable tears showed less favorable outcomes.	Open and arthroscopic repair provide equivalent outcomes; tendon healing is the key determinant of success.
Dickinson et al. (2019) [17]	55 patients (mean age: approximately 62 years)	Shoulder Pain and Disability Index (SPADI)	Physical Therapy Group: Formal physiotherapy including rotator cuff strengthening, scapular stabilization, stretching exercises, and home exercise program. Natural History Group: No formal physical therapy during the first three months of nonoperative management.	Patients who received physical therapy within the first three months experienced significantly greater improvements in pain and function, as reflected by better SPADI scores at the three-month follow-up (p = 0.02), compared with those who did not receive physical therapy. However, these differences were not maintained over time, with no significant between-group differences observed at 6, 12, or 18 months. Additionally, improvements in pain and function increased with the number of therapy sessions up to approximately 16 sessions, after which further gains plateaued.	Early physical therapy provides short-term benefit, but long-term outcomes are similar to natural history.
Akbaba et al. (2019) [18]	40 patients (mean age: 48-50 years)	VAS, Disability of the Arm, Shoulder and Hand (DASH), SPADI, ROM, Pressure Pain Threshold (PPT)	Experimental Group: Standard physiotherapy program + trigger point treatment. Control Group: Standard physiotherapy program + sham trigger point treatment.	Both groups improved in pain, function, disability, and ROM; however, the trigger point treatment group showed significantly greater improvements in pain relief, functional outcomes, and pressure pain threshold, indicating that trigger point therapy enhanced the effects of conventional physiotherapy.	Trigger point therapy is an effective adjunct to conventional physiotherapy in rotator cuff pathology.
Şahin et al. (2021) [19]	60 patients (mean age: 57.3 ± 8.4 years, knot-tying; 56.4 ± 7.9 years, knotless)	CMS, American Shoulder and Elbow Surgeons (ASES), VAS, MRI integrity	Knot-Tying Group: Arthroscopic knot-tying suture-bridge rotator cuff repair. Knotless Group: Arthroscopic knotless suture-bridge rotator cuff repair.	Both knot-tying and knotless suture-bridge arthroscopic rotator cuff repair techniques resulted in significant improvements in pain and shoulder function, with comparable functional outcomes and overall retear rates (19.0% vs. 28.6%). However, the knot-tying technique was associated with a higher incidence of medial (type II) retears, while failures in the knotless group occurred more commonly at the tendon-bone interface.	Both techniques are effective, but knotless repair may reduce the risk of medial cuff failure.
Chang et al. (2022) [20]	115 planned participants (mean age: >50 years)	SST, Numeric Rating Scale (NRS), ROM, DASH, SPADI, EuroQol-5D-5L (EQ-5D-5L)	Digital Group: Augmented reality (AR)-based digital healthcare rehabilitation system from postoperative week 6-12. Conventional Group: Standard brochure-based home rehabilitation program.	No results were reported in this article because it is a study protocol. The trial was designed to evaluate whether an augmented reality-based digital rehabilitation system could improve postoperative shoulder function, pain, ROM, strength, and QOL compared with conventional home-based rehabilitation after rotator cuff repair.	Designed to evaluate the effectiveness of digital healthcare rehabilitation after rotator cuff repair.
Ponce-Fuentes et al. (2026) [21]	23 patients (mean age: 58.1± 6.6 years)	PPT, conditioned pain modulation (CPM), VAS, SPADI, WORC, Tampa Scale for kinesiophobia	Control intervention: Low-intensity shoulder isometric exercises alone. Experimental intervention: Low-intensity shoulder isometric exercises combined with blood flow restriction.	A single session of low-intensity isometric exercise with blood flow restriction resulted in only modest increases in pressure pain threshold, with no significant or clinically meaningful advantage over isometric exercise alone. Pain intensity remained similar between interventions, while isometric exercise alone produced comparable or, in some cases, greater improvements in conditioned pain modulation, indicating that blood flow restriction did not provide additional short-term pain-relieving benefits during rotator cuff repair rehabilitation.	Blood flow restriction did not provide additional hypoalgesic benefits during early rotator cuff repair rehabilitation.
Reza et al. (2026) [22]	59 enrolled, 55 analyzed (mean age: 44.93 ± 9.65 years)	NRS, SPADI	Ultrasound-guided dry needling of the affected rotator cuff tendon. Clinical assessments performed at 6 weeks, 12 weeks, and 24 weeks	Patients demonstrated significant reductions in pain and disability following ultrasound-guided dry needling. Mean NRS pain scores decreased from 7.07 ± 0.87 at baseline to 2.22 ± 0.65 at 24 weeks, while SPADI scores improved from 73.58 ± 5.95 to 24.76 ± 6.13 over the same period. Improvements were statistically significant at all follow-up assessments (p < 0.001). The supraspinatus tendon was the most commonly involved tendon.	Ultrasound-guided dry needling improved pain and shoulder function in rotator cuff tendinopathy.
Yilmaz Muluk et al. (2026) [23]	90 patients (mean age: 51.19 ± 7.07 years)	Primary outcomes: VAS for pain, Quick Disabilities of the Arm, Shoulder and Hand (Quick DASH) score. Secondary outcomes: Exercise adherence, patient satisfaction, travel distance and time burden	Telemedicine Group: Asynchronous exercise videos with remote video-call follow-up. Control Group: Illustrated exercise brochure with conventional in-person follow-up.	Both the telemedicine and control groups showed significant improvements in pain and function, with VAS and Quick DASH scores improving significantly from baseline (p < 0.001) and no significant differences between groups in clinical outcomes. However, patients in the telemedicine group demonstrated higher exercise adherence and greater satisfaction while reducing travel time and distance, indicating that telemedicine was as effective as conventional follow-up for rotator cuff rehabilitation.	Telemedicine is a non-inferior and practical alternative to conventional rehabilitation for rotator cuff syndrome.

Risk of Bias Assessment

The risk of bias assessment revealed a predominantly high standard of methodological quality across the included literature. The majority of included RCTs, specifically Carr et al. (2017), Şahin et al. (2021), Akbaba et al. (2019), and Ponce-Fuentes et al. (2026), achieved an overall "low" risk of bias rating across all five RoB 2 domains, reflecting robust and adequately described randomization procedures, complete and unbiased outcome data reporting, and objective standardized measurement approaches. A rating of "some concern" was assigned to Kukkonen et al. (2015), Lambers Heerspink et al. (2015), Baumgarten et al. (2016), Chang et al. (2022), and Yilmaz Muluk et al. (2026). In each case, the primary source of concern was a "high" risk in the "deviations from intended interventions" domain, reflecting an inherent and methodologically unavoidable limitation of comparative trials involving surgical versus conservative or exercise-based interventions, where blinding of participants and treating clinicians is not feasible. Consequently, these studies were judged to have a high risk of bias in this domain despite otherwise robust methodological quality. Reza et al. (2026) received an "unclear" rating for the randomization domain attributable to insufficient detail regarding allocation concealment methodology. The sole observational cohort study (Dickinson et al., 2019), assessed using the ROBINS-I tool, received a "moderate" overall risk of bias rating, reflecting the known limitations of retrospective non-randomized designs in achieving adequate confounding adjustment. Table 2 presents the results of the risk of bias assessment for 11 included RCTs using the RoB 2 tool (Cochrane Collaboration).

**Table 2:** Risk of bias assessment for 11 included RCTs using the RoB 2 tool (Cochrane Collaboration). RCT: randomized controlled trial; RoB: risk of bias

Study	Randomization process	Deviations from interventions	Missing outcome data	Measurement of outcome	Selection of reported result	Overall risk
Kukkonen et al. (2015) [12]	Low	High	Low	Low	Low	Some concern
Lambers Heerspink et al. (2015) [13]	Low	High	Low	Low	Low	Some concern
Analan et al. (2015) [14]	Low	Low	Low	Low	Low	Some concern
Baumgarten et al. (2016) [15]	Low	High	Low	Low	Low	Some concern
Carr et al. (2017) [16]	Low	Low	Low	Low	Low	Low
Akbaba et al. (2019) [18]	Low	Low	Low	Low	Low	Low
Sahin et al. (2021) [19]	Low	Low	Low	Low	Low	Low
Chang et al. (2022) [20]	Low	High	Low	Low	Low	Some concern
Ponce-Fuentes et al. (2026) [21]	Low	Low	Low	Low	Low	Low
Reza et al. (2026) [22]	Unclear	High	Low	Low	Low	Some concern
Yilmaz Muluk et al. (2026) [23]	Low	High	Low	Low	Low	Some concern

Table 3 presents the risk of bias assessment for the non-randomized observational study using the ROBINS-I tool.

**Table 3:** Risk of bias assessment for the non-randomized observational study using the ROBINS-I tool. ROBINS-1 tool: Risk of Bias-I tool.

Study	Bias due to confounding	Bias in participant selection	Bias in the classification of Interventions	Bias due to deviations from interventions	Bias due to missing data	Bias in the measurement of outcomes	Bias in the selection of reported results	Overall risk
Dickinson et al. (2019) [17]	Moderate	Low	Low	Low	Low	Low	Moderate	Moderate

 Discussion

This systematic review synthesizes evidence from 12 methodologically rigorous studies, yielding several convergent and clinically actionable conclusions regarding the full spectrum of rotator cuff failure management. The overarching finding is that the management of rotator cuff pathology demands a nuanced, individualized, and milestone-driven approach - one that simultaneously acknowledges the progressive biological nature of the disease, respects the finite window of structural reparability, and leverages the increasingly sophisticated diagnostic and therapeutic tools available to the modern clinician.

Surgical Approaches and Their Central Role in Rotator Cuff Repair Failure

A comprehensive understanding of rotator cuff repair failure must begin with a rigorous examination of the surgical approach itself. The selection of operative technique, including the choice between open, mini-open, and arthroscopic repair, as well as suture anchor configuration and fixation strategy, is far from a matter of mere technical preference. Rather, it carries direct and measurable consequences for perioperative morbidity, tendon-healing biology, mechanical fixation strength, and long-term structural integrity. Carr et al. (2017), the landmark UKUFF trial established that modern arthroscopic repair achieves functional outcomes equivalent to traditional open techniques but with substantially reduced surgical morbidity and fewer complications [16]. This supports a definitive clinical shift toward arthroscopic intervention as the contemporary standard of care, optimizing early recovery without compromising long-term joint function [16]. 

However, the technical execution of arthroscopic rotator cuff repair carries its own distinct spectrum of failure mechanisms that surgeons and rehabilitation specialists must understand in detail to optimize outcomes. Suture anchor configuration, particularly the choice between knot-tying and knotless suture-bridge repair, exerts a significant and measurable influence on the pattern and location of structural failure following repair. Şahin et al. (2021) demonstrated, across two prospective randomized trials (combined n = 192), that while both knot-tying and knotless suture-bridge configurations yielded equivalent short-term pain relief and functional scores, prospective data revealed that knot-tying techniques significantly increase the risk of medial-row (type II) retears [19]. Biomechanically, this suggests that knot sites introduce focal stress concentrations [19]. Clinically, surgeons should favor knotless configurations to distribute tension uniformly across the footprint, particularly in patients with compromised tendon tissue [19].

Biological factors at the tendon-to-bone repair interface represent some of the most critical and least modifiable determinants of surgical success. Tendon-to-bone integration following rotator cuff repair is a prolonged biological process that unfolds over weeks to months and progresses through the recognized phases of inflammation, proliferation, and remodeling. The ultimate structural outcome is profoundly influenced by both the preoperative quality of the rotator cuff tissue and the biological milieu of the healing environment. Advanced fatty infiltration of the rotator cuff musculature, classified as Goutallier Grades 3-4, and severe muscle atrophy demonstrated by a positive tangential sign are among the strongest independent predictors of structural repair failure because they reflect irreversible compromise of the cellular machinery and mechanical substrate required for tendon-to-bone integration [4,5]. This biological reality lends considerable urgency to the concept of timely intervention. Each month of delay during which fatty infiltration progresses beyond the critical Goutallier Grade 2 threshold may transform a structurally reparable tear into a biologically compromised lesion with a substantially elevated risk of retear.

Patient-specific biological factors further modulate the healing response and influence surgical outcomes. Advanced age, particularly beyond 65 years, type 2 diabetes mellitus, active tobacco smoking, hypercholesterolemia, and chronic systemic corticosteroid use have each been shown to impair tendon vascularity and cellular healing capacity, thereby increasing the probability of repair failure even after technically successful anatomic reconstruction [4,5]. The morphological characteristics of the tear at the time of surgery, including anteroposterior and mediolateral tear dimensions, degree of tendon retraction, number of tendons involved, and quality of the residual tissue, also exert substantial independent prognostic influence on repair durability [3]. Massive tears involving two or more tendons, particularly those characterized by marked medial retraction and combined supraspinatus-infraspinatus involvement, demonstrate significantly higher rates of structural failure despite technically successful repair [8].

Postoperative management and adherence to rehabilitation protocols are equally important determinants of surgical success and should be regarded as integral components of the treatment plan rather than adjunctive considerations. Premature or excessive mechanical loading during the critical early tendon-to-bone healing phase, noncompliance with prescribed range-of-motion restrictions, and inadequate participation in structured progressive rehabilitation have each been associated with an increased biomechanical risk of repair failure [8]. The landmark RCT conducted by Baumgarten et al. (2016) demonstrated that introducing structured pulley exercises at six weeks post-repair safely accelerates range-of-motion recovery without increasing re-tear rates or compromising construct integrity [15]. This confirms that milestone-driven, progressively loaded rehabilitation protocols can optimize early movement while adhering strictly to biological tendon healing timelines [15].

Conservative Management and the Critical Therapeutic Threshold

For chronic, non-traumatic, degenerative RTs in appropriately selected patients, typically older individuals with lower functional demands and small-to-medium tear sizes, the synthesized evidence from Kukkonen et al. (2015) and Lambers Heerspink et al. (2015) provides a robust and clinically meaningful foundation for structured conservative management as the preferred initial treatment strategy [12,13]. At 12 to 24 months of follow-up, structured physical therapy produced improvements in Constant and DASH scores that were statistically equivalent to those achieved with surgical repair in this patient population. Lambers Heerspink et al. reported a non-significant mean between-group difference in Constant Score of only 2.3 points in favor of surgery (p = 0.441) [13]. Collectively, these findings support conservative management as a defensible, evidence-based first-line intervention for carefully selected patients with degenerative rotator cuff pathology [12,13].

However, the longitudinal cohort data reported by Dickinson et al. (2019) introduce an important clinical consideration regarding the limitations of prolonged conservative management. Their prospective findings suggest a dose-response threshold at approximately 16 structured physical therapy sessions, beyond which functional improvements appear to plateau and additional gains become limited [17]. Furthermore, the functional advantage of early physical therapy over the natural history of the condition, evident and statistically significant at the three-month assessment, was no longer apparent at 18 months, with long-term outcomes becoming statistically equivalent between treated and untreated patient cohorts [17].

These findings highlight the importance of ongoing clinical monitoring during conservative management. Conservative treatment should not be viewed as an indefinite strategy in patients who demonstrate persistent symptoms, functional limitations, or evidence of progressive structural deterioration. Patients who fail to achieve predefined functional milestones, such as meaningful reductions in pain scores, improvements in SPADI or Constant Score outcomes, and restoration of functional range of motion after approximately 16 structured physical therapy sessions, should undergo reassessment to determine whether surgical intervention may be appropriate. Such timely evaluation may help prevent further progression of fatty infiltration and muscle atrophy, which are known to adversely affect tendon reparability and long-term treatment outcomes [17].

Emerging Rehabilitation Adjuncts and Their Evidence Base

The optimization of both postoperative and conservative rehabilitation has been advanced by several adjunctive interventions that have been evaluated in recent clinical studies. Blood flow restriction (BFR) training, investigated by Ponce-Fuentes et al. (2026) in a randomized crossover trial, was found to be safe and feasible during the early phases of rotator cuff rehabilitation. However, it did not demonstrate clinically meaningful additional hypoalgesic benefits compared with low-intensity isometric exercise alone, suggesting that its role in postoperative rehabilitation requires further investigation [21].

Ultrasound-guided dry needling, evaluated by Reza et al. (2026), was associated with significant improvements in pain intensity and functional disability, as reflected by reductions in NRS and SPADI scores [22]. These findings suggest that dry needling may serve as a useful adjunct to conventional rehabilitation programs in patients with rotator cuff pathology.

Digital telemedicine-delivered rehabilitation, assessed by Yilmaz Muluk et al. (2026) in an RCT, demonstrated pain and functional outcomes comparable to those achieved with conventional in-person rehabilitation. Furthermore, patients in the telemedicine group exhibited higher exercise adherence, greater satisfaction, and reduced travel burden. These findings suggest that telemedicine-based rehabilitation may represent a practical, scalable, and patient-centered approach to overcoming barriers related to geographic accessibility, transportation requirements, and long-term treatment adherence [23].

Synthesis and Clinical Implications

The synthesis of findings across all 12 included studies yields a coherent, evidence-grounded, and immediately actionable clinical framework for rotator cuff failure management. For chronic degenerative tears in appropriately selected patients, structured physical therapy constitutes an evidence-supported first-line intervention, with objective functional milestones assessed at regular intervals and a clear clinical timeline beyond which further conservative treatment should not delay surgical referral if progress plateaus. For acute traumatic tears, high-demand active patients, athletes, and those demonstrating early biological compromise with Goutallier Grade 2 or higher fatty infiltration, early arthroscopic repair - executed with awareness of the patient-specific biological and mechanical factors that mediate structural healing - offers the optimal window for anatomic repair and durable functional restoration. Postoperatively, evidence-based progressive loading protocols, adjunctive pain modulation strategies, and digital rehabilitation delivery platforms should be systematically integrated to maximize adherence, accelerate functional recovery, and minimize retear risk.

Challenges

Managing rotator cuff failure presents complex, multifaceted, and persistent clinical challenges that extend across the full care continuum. The most fundamental and pervasive challenge is the profound - and frequently paradoxical - disconnect between radiographic severity and patient-reported functional capacity: patients with imaging-confirmed large full-thickness tears may maintain near-normal shoulder function for extended periods, while others with comparatively smaller tears experience profound and disabling pain and disability. This fundamental radiological-clinical dissociation severely complicates prognostication, creates diagnostic uncertainty regarding optimal intervention timing, and challenges clinicians' capacity to identify the precise moment at which structural reparability will be irreversibly compromised.

The inherent methodological challenge of blinding participants, treating surgeons, and outcome assessors in comparative trials involving surgical versus conservative interventions introduces unavoidable performance and detection biases that limit the internal validity of existing head-to-head comparative effectiveness studies - a limitation reflected in the "some concern" risk of bias ratings assigned to several included trials. Standardized, threshold-based clinical decision algorithms integrating preoperative biological markers (Goutallier grade, muscle cross-sectional area, tendon retraction measurement) with patient-specific functional and demand profiles do not currently exist in a validated form. Furthermore, systemic health system barriers, including geographic access to specialized musculoskeletal physical therapy, financial costs of prolonged conservative care in resource-limited settings, and variable patient adherence across extended rehabilitation courses, disproportionately impact outcomes in underserved populations.

Future Directions

Future research efforts must be strategically prioritized toward the development and validation of evidence-based, threshold-defined clinical decision algorithms that integrate biological imaging biomarkers, including Goutallier fatty infiltration grade, muscle cross-sectional area on quantitative MRI, and objective tendon quality indices with patient-level functional profiles and activity demand assessments. Such validated algorithms would provide clinicians with precise, individualized tools for evidence-based identification of the optimal surgical intervention window, personalizing the transition from conservative to operative management and maximizing the probability of durable anatomical repair.

Dynamic assessment methodologies, including real-time high-resolution musculoskeletal ultrasonography combined with three-dimensional kinematic mapping of active shoulder motion, hold significant promise for characterizing tendon translation dynamics and force couple efficiency in ways that static imaging modalities fundamentally cannot. Artificial intelligence-driven automated image analysis for Goutallier grade quantification, muscle volume measurement, and retear risk stratification represents an imminent and high-impact application of machine learning to clinical decision support in rotator cuff management. Longitudinal multicenter RCTs incorporating standardized imaging endpoints (including serial MRI for fatty infiltration and muscle atrophy quantification), a minimum five-year structured follow-up, and comprehensive biological sampling will be essential to definitively characterize the natural history of rotator cuff failure and rigorously establish the long-term structural and functional durability of contemporary repair techniques and emerging biological augmentation strategies.

## Conclusions

Rotator cuff failure represents a mechanistically complex, biologically progressive, and clinically consequential musculoskeletal condition in which the consequences of delayed, inadequate, or misdirected intervention may be irreversible. This systematic review synthesizes evidence across the full continuum of management from the intrinsic biological mechanisms of tendon degeneration and the cascading biomechanical sequelae of tear progression, through the comparative efficacy of conservative and surgical treatment strategies, to the expanding evidence base supporting advanced rehabilitation adjuncts and digital delivery platforms.

The central and clinically urgent message of this synthesis is one of informed precision: while structured physical therapy provides equivalent long-term functional outcomes to surgery for appropriately selected patients with chronic degenerative tears, the evidence suggests that nonoperative progress typically plateaus after an initial course of structured rehabilitation. This observed trajectory demands active clinical monitoring and a well-timed transition to surgical evaluation if predefined functional milestones are not achieved. For acute tears, high-demand patients, and those demonstrating early biological compromise, arthroscopic repair selected and executed with comprehensive awareness of the patient-specific biological and mechanical determinants of structural healing offers the greatest probability of durable functional restoration. Surgical approach selection, suture anchor technique, preoperative tissue biology, and postoperative rehabilitation protocol adherence are each independently determinative of repair success, and each must be addressed within a coherent, individualized, and milestone-tracked management framework. The systematic integration of evidence-based rehabilitation adjuncts, including blood flow restriction, ultrasound-guided dry needling, and asynchronous telemedicine delivery and the continued development of AI-driven predictive decision tools will progressively advance the precision, personalization, and equity of rotator cuff failure management in the years ahead.
